# *Luteodorsum huanglongense* (Gomphaceae, Gomphales), a New Genus and Species of Gomphoid Fungus from the Loess Plateau, Northwest China

**DOI:** 10.3390/jof9060664

**Published:** 2023-06-13

**Authors:** Zijia Peng, Yiming Wu, Zeyu Luo, Chaowei Xiong, Xiaoyong Liu, Bin Wang, Baoyou Ma, Jianxian Wei, Zhongdong Yu

**Affiliations:** 1 College of Forestry, Northwest A & F University, Xianyang 712100, China; 2 College of Life Sciences, Shandong Normal University, Jinan 250358, China; 3 State-Owned Forest Administration Bureau of Huanglong Mountains, Yan’an 715700, China; 4 Administration Bureau of Huanglong Mountains Crossoptilon mantchuricum National Nature Reserve, Yan’an 715700, China

**Keywords:** Agaricomycetes, Basidiomycota, EDS, Gomphaceae, systematics, taxonomy

## Abstract

During an investigation of the macrofungal flora in the Huanglong Mountains of the Loess Plateau, northwest China, a unique gomphoid fungus was discovered and collected. After morphological identification and molecular phylogenetic analyses, a new genus named *Luteodorsum* and its type species, *L. huanglongense*, were proposed. Phylogenetic analyses were conducted using datasets of nuclear ribosomal DNA 28S large subunit (LSU), mitochondrial (mt) adenosine triphosphatase (ATPase) subunit 6 (atp6), and mt small-subunit rDNA (mtSSU). The results confirmed that *L. huanglongense* forms an independent clade within Gomphales, with full maximum likelihood bootstrap support (MLBS), maximum parsimony bootstrap support (MPBS), and Bayesian posterior probability (BPP). *L. huanglongense* is characterized by its sandy-brown, orange-brown, or coffee-brown color; clavate to infundibuliform shape; wrinkled and ridged hymenophore; ellipsoid to obovoid warted basidiospores; cylindrical to clavate flexuous pleurocystidia; and crystal basal mycelium. Overall, this study contributes to the growing body of knowledge on the diversity and evolution of Gomphales and provides valuable insights into the unique fungal flora found in the Huanglong Mountains.

## 1. Introduction

The order Gomphales Jülich (Agaricomycetes, Basidiomycota) is regarded as a monophyletic group that is closely related to the Geastrales, Phallales, Gloeophyllales, and Hysterangiales orders [1,2,3]. It comprises over 410 species within 3 families, namely Clavariadelphaceae Corner, Lentariaceae Jülich, and Gomphaceae Donk [3,4]. These species are widely distributed worldwide, particularly in the northern hemisphere, and are mostly mycorrhizal or saprotrophic, playing important roles in fungal diversity and forest ecology [2,3,5].

However, the three families of Gomphales exhibit significant macromorphological differentiation. Clavariadelphaceae are typified by club-shaped (clavaroid) basidiomes, such as *Clavariadelphus* Donk, or stalked basidiomes with teeth underneath the cap, such as the genus *Beenakia* D.A. Reid [6,7,8]. Lentariaceae are characterized by stalked clavarioid basidiomes, such as *Lentaria* Corner, or resupinate-hydnoid basidiomes, such as *Hydnocristella* R.H. Petersen and *Kavinia* Pilát [9,10,11]. Gomphaceae, which encompass 15 genera, exhibit the most differentiated morphologies among Gomphales, with hypogeous or epigeous; solitary or gregarious; infundibuliform; coralliform; and clavate or irregularly branched forms [3,4,12]. For instance, *Ramaria* Fr. ex Bonord. spp. are coral fungi, while *Gloeocantharellus* Singer and *Gomphocantharellus* L. Fan, Y.Y. Xu, Zhu L. Yang, and S.P. Jian spp. are gilled mushrooms, and *Gautieria* Vittad. spp. are false truffles. Furthermore, *Gomphus* Pers., *Phaeoclavulina* Brinkmann, and *Turbinellus* Earle spp. are cantharelloid–gomphoid [1,4].

In addition to ecological, molecular phylogenetic, and macromorphological evidence, microscopic and ultramicroscopic features are of great taxonomic value. For Gomphales, comparative descriptions of the different types of spore ornamentation and hilar appendices have helped taxonomists propose relationships between the species, genus, and family levels over recent decades [4,8,13,14,15,16,17,18]. The special gloeocystidia in the hymenium make *Gloeocantharellus* a recognizable genus of Gomphaceae [19]. Moreover, variations in the basal mycelium, rhizomorphs, and crystals on the surface are also helpful in distinguishing several Clavariadelphus, Lentariaceae, *Gomphocantharellus*, and *Phaeoclavulina* species of Gomphales [4,8,9,10,20].

During a survey of the macrofungi flora in the Huanglong Mountains *Crossoptilon mantchuricum* National Nature Reserve located in the Loess Plateau of northwest China, basidiomes forming a conspicuous fairy ring on litter in a mixed broadleaf–conifer forest were noticed. Although this species resembled a gomphoid mushroom, it was distinct from any known species. After elaborative morphological observations and phylogenetic analyses, a new genus of *Luteodorsum* and its type species *L. huanglongense* sp. nov. were proposed.

## 2. Materials and Methods

### 2.1. Morphological Studies

Specimens were collected and photographed from the Huanglong Mountains *Crossoptilon mantchuricum* National Nature Reserve in Shaanxi Province, China. After being dried, voucher specimens were deposited at Herbarium Mycologicum Academiae Sinicae (HMAS), Institute of Microbiology, Chinese Academy of Sciences, Beijing, China, and the Mycological Herbarium of the Forestry College, Northwest A & F University (HMNWAFU-CF), Shaanxi Province, China. Macroscopic characteristics were recorded from both fresh and dried specimens, and standardized color-code designations matching the color of the description were taken from Color-hex (https://www.color-hex.com/, accessed on 3 May 2023). Microscopic observations followed Xu et al. [4]. Fungal histological sections of dried specimens were mounted in 3% KOH, Congo red, Melzer’s reagent [21], and 0.1% (*w*/*v*) Cotton blue in lactic acid and observed under an Olympus CX41RF microscope (Tokyo, Japan). The notation “[n/m/p]” indicates n basidiospores from m basidiomes of p collections. The dimensions of the basidiospores are presented using notation of the form (a–)b–c(–d). The range b–c contains a minimum of 90% of the measured values. Extreme values, i.e., a and d, are presented in parentheses. L_m_ and W_m_ indicate the average basidiospore length and width (±standard deviation) for the measured basidiospores, respectively. Q represents the mean length/width ratio of a basidiospore from the side view, and Q_m_ represents the average Q of all specimens ± sample standard deviation. Hand-drawn illustrations of the microscopic features were produced using a digital pen tablet (GAOMON WH850) and Adobe Photoshop 2022 software, as previously described [22]. To observe the ultrastructure, basidiospores and basal mycelium scraped from dried specimens were mounted on a scanning electron microscopy (SEM) stub with doubled-sided carbon tape, coated with platinum film using a Shinkuu MSP-1S ion-sputter coater (Mito, Japan), and examined and photographed with a Hitachi S-4800 SEM (Tokyo, Japan). Qualitative X-ray microanalyses were performed on crystals using an energy-dispersive X-ray spectrometry (EDS) microprobe that was fitted on the same SEM and processed using EDAX Genesis Spectrum v6.29 software.

### 2.2. DNA Extraction, PCR Amplification, and DNA Sequencing

Small amounts of dried basidiome tissues were collected to extract the total genomic DNA using a rapid fungi genomic DNA isolation kit (Sangon Biotech, Shanghai, China). Polymerase chain reaction (PCR) amplification was performed for mitochondrial (mt) adenosine triphosphatase (ATPase) subunit 6 (atp6) using primers ATP6-1/ATP6-2, mt ribosomal DNA small subunit (mtSSU) using primers MS1/MS2, nuclear ribosomal DNA internal transcribed spacer (ITS) region using primers ITS1/ITS4, and nuclear ribosomal DNA 28S large subunit (LSU) using primers LR0R/LR3 [23,24,25].

PCR was performed in a 20 μL reaction volume comprising 2 μL of the DNA template; 1 μL of each primer (10 μM); 10 μL 2× Taq PCR Master Mix (Cowin Biotech, Taizhou, China); and 6 µL ddH_2_O. PCR amplification procedures were performed using a GeneAmp PCR TC-96 (Bioer Technology, Hangzhou, China) according to the following conditions: for atp6, an initial denaturation stage at 94 °C for 3 min, followed by 35 cycles of denaturation at 94 °C for 30 s, annealing at 43 °C for 45 s, extension at 72 °C for 1 min, and a final extension at 72 °C for 10 min, with the procedure ending at 4 °C; for mtSSU, ITS, and LSU, the annealing temperatures were 45 °C, 57 °C, and 50 °C, separately. PCR products were separated via electrophoresis on a 1% agarose gel in a 1× TAE buffer (Solarbio, Beijing, China) and then sequenced by Sangon Biotech Co., Ltd. (Shanghai, China).

### 2.3. Phylogenetic Analyses

To determine the phylogenetic position of the new genus and species within Gomphales, phylogenetic analyses were conducted using three independent loci of LSU, atp6, and mtSSU based on the maximum likelihood (ML), maximum parsimony (MP), and Bayesian inference (BI) [1,2,4]. The LSU, atp6, and mtSSU sequences of the reference taxa were aligned using the MUSCLE algorithm and manually modified in MEGA-X, respectively, and then combined in SequenceMatrix 1.8 [26]. Three taxa of *Mutinus elegans* (Mont.) E. Fisch., *Phallus impudicus* L., and *Pseudocolus fusiformis* (E. Fisch.) Lloyd were selected as outgroups, as in previous studies [2,4,27].

The ML analysis was conducted in raxmlGUI 2.0 [28] using a GTRGAMMAI model, with all other parameters set to default. A total of 1000 bootstrap replicates were computed using a rapid bootstrap analysis and search for the best-scoring ML tree. The MP analysis was carried out in PAUP* 4.0a169, and bootstrap values were generated with 1000 replicate searches on all parsimony-informative characteristics using 100 random sequence addition replications [29]. Tree bisection reconnection (TBR) branch-swapping algorithms were employed. Tree length (TL), the consistency index (CI), the retention index (RI), the rescaled consistency index (RC), and the homoplasy index (HI) were also calculated. BI analysis was performed in MrBayes 3.1.2 using a partitioned mixed model with LSU, atp6, and mtSSU sequences defined as three independent partitions [30]. Each gene was modeled separately with different parameters. The best-fitting substitution model for each gene was GTR + I + G according to MrModeltest 2.3 software. Four Markov chain Monte Carlo (MCMC) models were run for an initial 5,000,000 generations under the default settings, and continued with analysis until the average standard deviations of the split frequency (ASDSF) values were lower than 0.01 at the end of the runs. Trees were sampled every 100 generations after burn-in (25% of trees were discarded as the burn-in phase of the analyses, set up well after convergence), and 50% majority-rule consensus trees were constructed. Clades with bootstrap support (MLBS and MPBS) ≥ 70% and a Bayesian posterior probability (BPP) ≥ 0.95 were considered significantly supported [31,32]. Phylogenetic trees were viewed with FigTree v1.4.3 and manually annotated using Adobe Illustrator 2022 software.

## 3. Results

### 3.1. Phylogenetic Analyses

A total of 238 sequences were used for phylogenetic analyses, which consisted of 226 reference sequences of 81 related taxa downloaded from GenBank, as used in previous studies [2,4], as well as 12 new sequences (4 for LSU, 4 for atp6, and 4 for mtSSU) generated from voucher specimens collected in 2021 and 2022. Accession numbers for all newly generated sequences were obtained by submitting them to GenBank, and details of the sequences used for phylogenetic analyses are provided in Table 1. Additionally, four new ITS sequences were generated and submitted to GenBank. The atp6 dataset comprised 80 taxa and 722 characteristics, of with 240 were constant, 94 were variable and parsimony-uninformative, and 388 were variable and parsimony-informative. The mtSSU dataset comprised 77 taxa and 628 characteristics, of with 278 were constant, 135 were variable and parsimony-uninformative, and 215 were variable and parsimony-informative. The LSU dataset comprised 81 taxa and 714 characteristics, of with 337 were constant, 107 were variable and parsimony-uninformative, and 270 were variable and parsimony-informative. Furthermore, a combined atp6–mtSSU matrix with 1350 total characteristics and a combined LSU–atp6–mtSSU matrix with 2064 total characteristics were generated in this study.

Phylogenetic analyses based on a single gene were analyzed at first (Supplementary Figures S1–S3). The results of the atp6 phylogenetic tree confirmed the paraphyletic status of genus *Ramaria* and the monophyletic status of 11 other genera except the genus *Phaeoclavulina* (Supplementary Figure S1). Differently, our result split *Phaeoclavulina* into only two clades instead of three clades, as Xu et al. described [4]. The results of the mtSSU phylogenetic tree agreed with Xu et al. [4]. Ten genera were confirmed as monophyletic, but the monophyletic status of *Gloeocanantharellus* and *Clavariadelphus* were not supported with the existence of more than one branch. Meanwhile, for *Ramaria* subg. *Laeticolora*; *Ramaria* subg. *Ramaria*; and *Ramaria* subg, *Lentoramaria* were not distinguished well, and only *Ramaria* subg. *Echinoramaria* formed a relatively independent clade (Supplementary Figure S2). In the LSU phylogenetic tree, only nine genera within Gomphales were confirmed as monophyletic. The genus *Gautieria* was spilt into two clades, which never occurred in the two previous phylogenetic trees (Supplementary Figure S3). Although the topological structures were inconsistent when analyzing these three individual genes, all phylogenetic trees indicated that our specimens formed a completely stable and independent clade, thus being of a monophyletic status.

**Figure 1:**
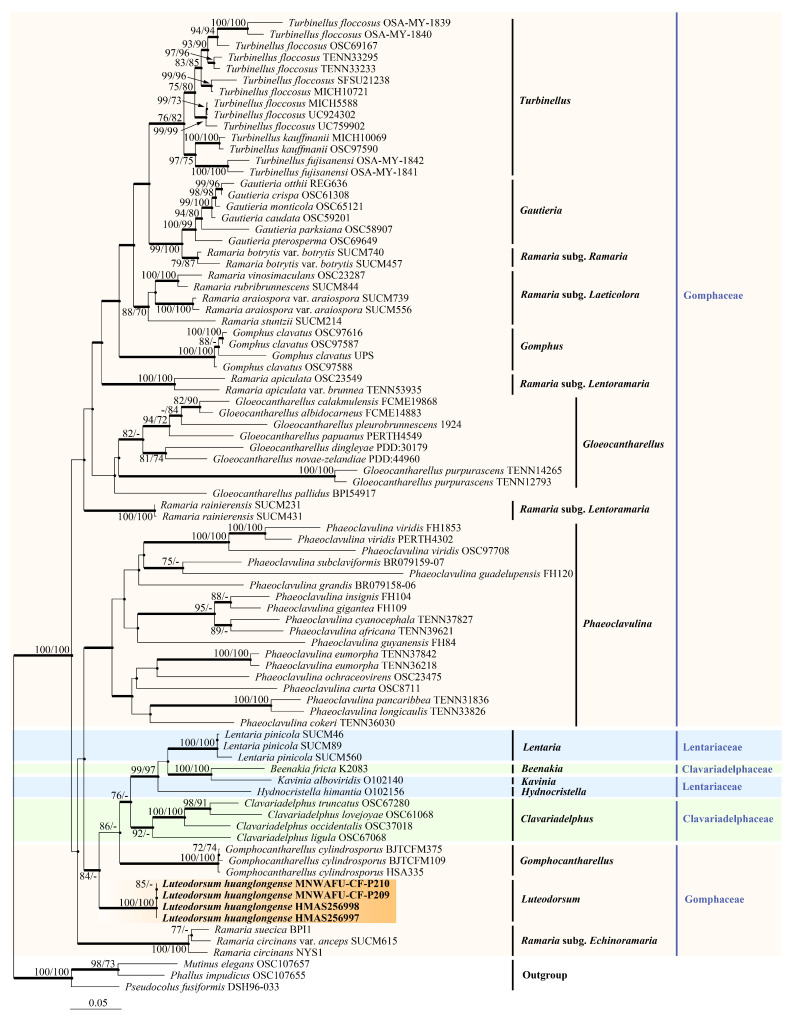
Phylogenetic tree generated from a maximum likelihood analysis based on combined LSU, atp6, and mtSSU sequences depicting the phylogenetic relationships of Gomphales. *Phallus impudicus*, *Mutinus elegans*, and *Pseudocolus fusiformis* were used as outgroups. The nodes above the branches indicate the maximum likelihood bootstrap support (MLBS) values (≥70%) and maximum parsimony bootstrap support (MPBS) values (≥70%). The branches that presented a Bayesian posterior probability (BPP) ≥ 0.95 are thicker. The novel sequences are highlighted in bold and an orange shade.

When combining and analyzing multiple loci, the four subgenera of the paraphyletic genus *Ramaria* were clearly distinguished, and all other genera were confirmed as monophyletic, in line with previous studies [2,4]. The tree topologies of atp6–mtSSU and LSU–atp6–mtSSU phylogenetic trees were almost identical (Supplementary Figure S4, Figure 1). The MP analysis of the combined atp6–mtSSU dataset resulted in a highly parsimonious tree with a TL of 3678 steps, a CI of 0.368, an RI of 0.659, a RC of 0.243, and an HI of 0.632. The MP analysis of the combined LSU–atp6–mtSSU dataset resulted in a highly parsimonious tree with a TL of 5596 steps, a CI of 0.355, an RI of 0.611, a RC of 0.217, and an HI of 0.645. The ML, MP, and BI analyses of combined datasets yielded very similar tree topologies with minimal variation in statistical support values, so only the tree inferred from the ML analysis is presented.

The phylogenetic tree revealed that the Gomphales sequences formed a distinct clade presenting significant support values (MLBS/MPBS/BPP = 100%/98%/1 in the atp6–mtSSU tree, and MLBS/MPBS/BPP = 100%/100%/1 in the LSU–atp6–mtSSU tree), with the sequences from northwest China being well-clustered within Gomphales (Supplementary Figure S4, Figure 1). Within the Gomphales phylogenetic tree, the Gomphaceae family contained seven genera, with *Ramaria* being paraphyletic and the other six genera being monophyletic. The Lentariaceae and Clavariadelphaceae families were paraphyletic and clustered in a clade with a high BPP of 0.99 and a moderate MLBS/MPBS of 68%/69% in the atp6–mtSSU tree (Supplementary Figure S4), as well as high MLBS/BPP values of 76%/0.97 and a moderate MPBS of 64% in the LSU–atp6–mtSSU tree (Figure 1). These results concurred with those of previous studies by Giachini et al. and Xu et al. [2,4]. Four specimens collected from the Loess Plateau of northwest China (HMAS256997, HMAS256998, MNWAFU-CF-P209, and MNWAFU-CF-P210) formed an independent clade with significantly strong support (MLBS = 100%, MPBS = 100%, BPP = 1) in both the atp6–mtSSU and LSU–atp6–mtSSU trees, and were grouped together with the taxa of *Gomphocantharellus*, Lentariaceae, and Clavariadelphaceae with significant MLBS/MPBS/BPP values of 80%/71%/1 in the atp6–mtSSU tree (Supplementary Figure S4), as well as significant MLBS/BPP values of 84%/1 and a moderate MPBS value of 65% in the LSU–atp6–mtSSU tree (Figure 1), respectively. Based on the phylogenetic tree, we proposed a novel genus, *Luteodorsum*, which had a closer phylogenetic relationship to *Gomphocantharellus* and the *Ramaria* subg. *Echinoramaria* of Gomphaceae, Lentariaceae, and Clavariadelphaceae than with other genera of Gomphaceae.

### 3.2. SEM Observation and Qualitative X-ray Microanalysis

**Figure 2:**
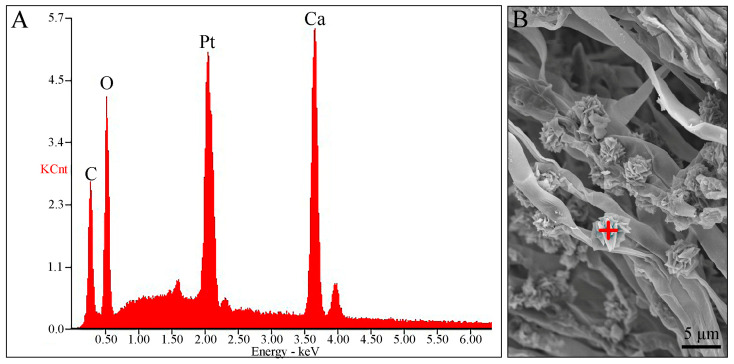
Qualitative X-ray microanalysis performed via energy-dispersive X-ray spectrometry (EDS). (**A**) EDS spectrum of crystals on the surface of the basal mycelium, (**B**) Spot-tested using EDS (red plus).

Several basidiospores and basal mycelia were scraped from dried specimens and photographed under SEM to observe their ultrastructure. Basidiospores were finely warted, and the basal mycelium was smooth. Interestingly, rosette-like crystals were observed above the hyphal surface of the basal mycelium (Figure 2B). To further understand the characteristics of this fungus, qualitative X-ray microanalysis was conducted using the same SEM to detect the crystal elements.

The results of the EDS spectrum showed four large X-ray peaks of calcium (Ca), platinum (Pt), carbon (C), and oxygen (O) (Figure 2A). The weight percentage and atomic percentage of the rosette-like crystals are listed in Table 2. The Pt was entirely derived from the ion-sputter coater and should be ignored. The crystals were confirmed to be calcium salt crystals, with the presence of only one metallic element. After removing the Pt, the corrected crystal weight comprised 60.90% Ca, 32.41% O, and 6.69% C, and the corrected crystal atoms comprised 37.04% Ca, 49.38% O, and 13.58% C, respectively.

### 3.3. Taxonomy

**Figure 3:**
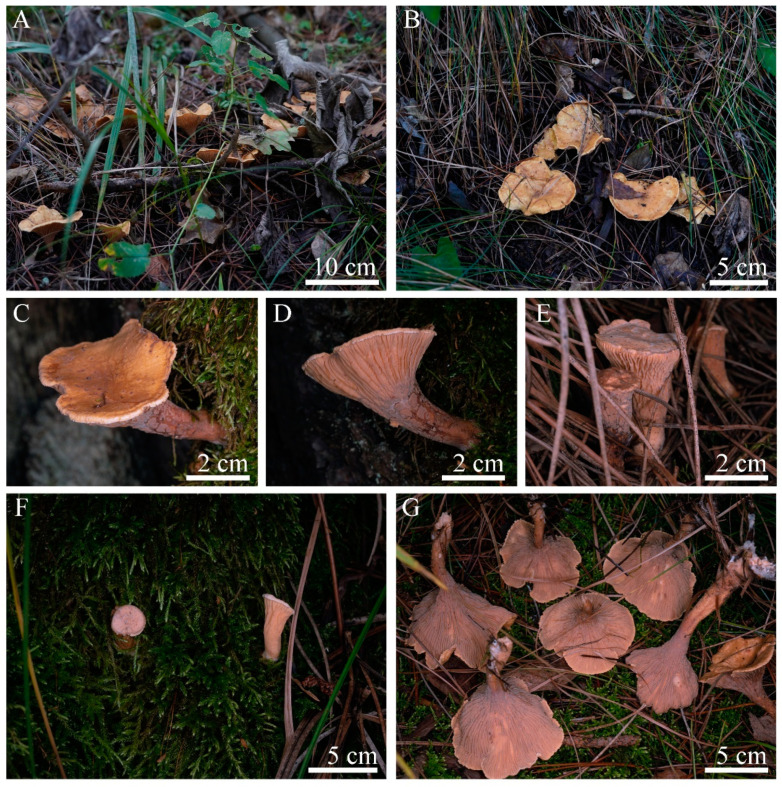
Habitat and basidiomes of *Luteodorsum huanglongense* (holotype HMAS256997). (**A**,**B**) Habitat in the field. (**C**–**F**) Young basidiomes. (**G**) Mature basidiomes.

**Figure 4:**
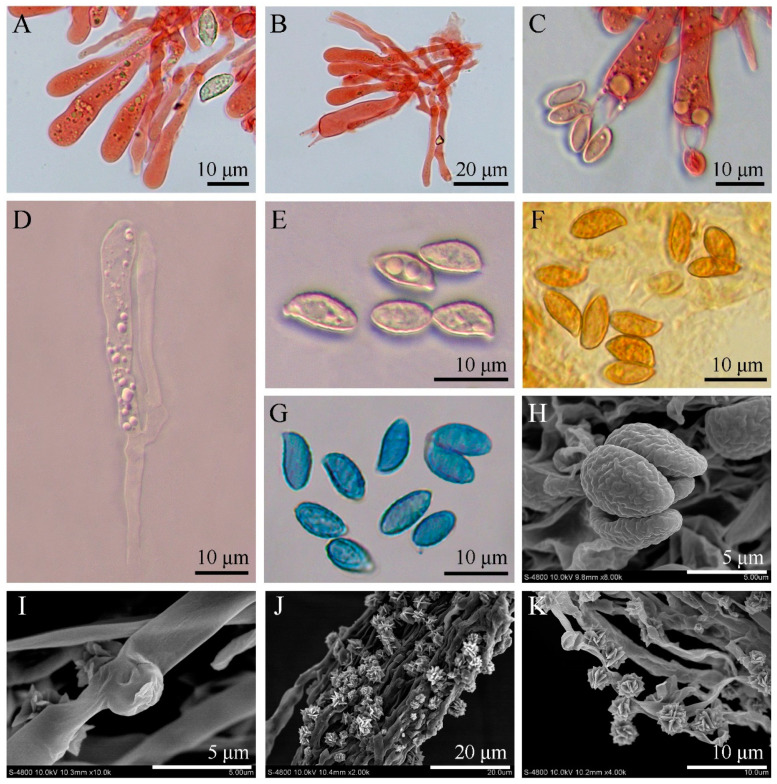
Microscopic and ultramicroscopic features of *Luteodorsum huanglongense*. (**A**) Pleurocystidia, basidioles, and basidiospores in Congo red. (**B**) Pleurocystidia, basidioles, and basidia in Congo red. (**C**) Basidia and basidiospores in Congo red. (**D**) Pleurocystidia and basidioles in KOH. (**E**) Basidiospores in KOH. (**F**) Basidiospores in Melzer’s reagent. (**G**) Basidiospores in Cotton blue. (**H**) Basidiospores under SEM observation. (**I**) Clamp connections of basal mycelium under SEM observation. (**J**,**K**) Basal mycelium and calcium salt crystals under SEM observation.

**Figure 5:**
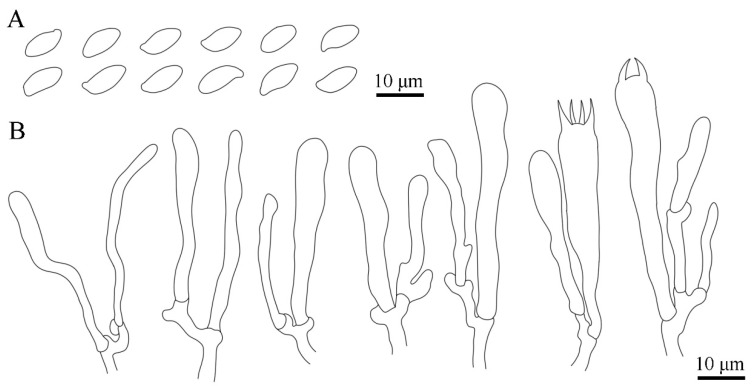
Microscopic features of *Luteodorsum huanglongense*. (**A**) Basidiospores. (**B**) Pleurocystidia, basidioles, and basidia.

***Luteodorsum*** Z.J. Peng, X.Y. Liu, & Z.D. Yu, **gen. nov.** (Figure 3, Figure 4 and Figure 5)

MycoBank 848312

Type species: *Luteodorsum huanglongense* Z.J. Peng, X.Y. Liu, and Z.D. Yu (described below).

Etymology: *Lute-* (Lat.), meaning yellow, in reference to the color of the dried hymenium; -*dorsum* (Lat.), derived from the wrinkled and ridge-like surface of the hymenium; *Luteodorsum* (Lat.), referring to the color and morphological similarity of the dried hymenium to the famous Loess Plateau of China, which is exactly the typical geomorphology of the city where the type species was collected.

Diagnosis: *Luteodorsum* differs from the five other cantharelloid–gomphoid genera of *Gomphocantharellus*, *Gloeocantharellus*, *Gomphus*, *Phaeoclavulina*, and *Turbinellus* due to its stipitate-pileate basidiomes presenting an almost glabrous to fibrillose pileus without obvious scales; its wrinkled, ridged, salmon to rosy-brown hymenophore; and its ellipsoid to obovoid warted basidiospores. Genetically, *Luteodorsum* forms a strongly autonomous well-clustered branch of Gomphales based on the LSU, atp6, and mtSSU sequences.

Description: *Basidiomes* stipitate-pileate, gomphoid, fleshy. *Pileus* clavate to horse-hoof-like at first, fan-shaped to funnel-shaped at maturity, surface coarse, almost glabrous to fibrillose, with sporadic warts, slightly hygrophanous, margin subundulate. *Hymenophore* decurrent; wrinkled and ridged; occasionally in irregular patches; light salmon, dark salmon, to rosy-brown; unchanging when exposed. *Stipe* central or slightly eccentric, cylindrical to slightly tapering downward, solid, with a white basal mycelial cord. *Pleurocystidia* scattered among and scarcely projecting beyond the basidia, cylindrical to clavate, flexuous, smooth. *Hyphae* with clamp connections. *Basel mycelium* smooth, with rosette-like druse crystals. *Basidiospores* ellipsoid to obovoid, ornamented with warts, light orange to light cinnamon, inamyloid, cyanophilic.

***Luteodorsum******huanglongense*** Z.J. Peng, X.Y. Liu, and Z.D. Yu, **sp. nov.** (Figure 3, Figure 4 and Figure 5)

MycoBank 848313

Typification: China, Shaanxi Province, Yan’an City, Huanglong County, Huanglong Mountains, Caijiachuan Forest Farm, on litter in mixed broadleaf–conifer forest dominated by *Pinus tabuliformis* Carrières [33], *Quercus mongolica* Fisch. ex Turcz. [34], and *Betula pendula* subsp. *mandshurica* (Regel) Ashburner and McAll. [35]; elev. 1330 m; 35°49′27″ N, 109°54′49″ E; 1 October 2021; Z.J. Peng, Z.Y. Luo, and Z.D. Yu, HL152 (holotype HMAS256997). GenBank: ITS = OQ801492; LSU = OQ801490; mtSSU = OQ801494; atp6 = OQ790052.

Etymology: *huanglongense* (Lat.), referring to the type locality in the Huanglong Mountains in Shaanxi Province, China.

Diagnosis: *L. huanglongense* differs from other species of gomphoid fungi due to the following combination of characteristics: basdiomes unipileate; pileus surface coarse; almost glabrous to fibrillose; slightly hygrophanous; sandy-brown, orange-brown to coffee-brown when fresh; cream, light yellow to fawn when drying out; hymenium occasionally in irregular patches; light salmon, dark salmon to rosy brown when fresh; wheat to earth yellow when drying out; basidiospores (8.5–)8.7–10.7(–12.4) × (4.1–)4.2–5.5(–5.9) μm with Q = 1.6–2.5, Q_m_ = 2.01 (±0.19); basidia 46–67 × 7–9 μm; pleurocystidia 34–49 × 2.7–5 μm, scattered among and scarcely projecting beyond the basidia; basal mycelium with rosette-like calcium salt crystals.

Description: *Basidiomes* erect; unipileate; fleshy when fresh; fragile when dry, solitary, scattered or in small groups. *Pileus* 4–12 mm wide; clavate to horse-hoof-like when young, then center depressed; fan-shaped to funnel-shaped (infundibuliform) at maturity; surface coarse; almost glabrous to fibrillose; slightly hygrophanous; sandy-brown (#d29459), orange-brown (#ab7257) to coffee-brown (#975c3c) when fresh; cream (#f9f2d8), light yellow (#f2e1b3) to fawn (#dfbc94) when drying out; margin subundulate; white to light orange (#d8ac93). *Hymenium* decurrent, with dichotomous, wrinkled, and ridged veins; occasionally in irregular patches; light salmon (#db9a86); dark salmon (#d08a71) to rosy-brown (#a07475) when fresh; wheat (#d8c6ae) to earth yellow (#ad7f44) when drying out. *Stipe* 27–43 × 5–14 mm, central or slightly eccentric, cylindrical to slightly tapering downward, solid, almost oncolorous with pileus, with white basal mycelial cord. Odor not distinctive, taste not recorded.

*Basidiospores* (8.5–)8.7–10.7(–12.4) × (4.1–)4.2–5.5(–5.9) μm, L_m_ × W_m_ = 9.7 (±0.62) × 4.8 (±0.39) μm, Q = 1.6–2.5, Q_m_ = 2.01 (±0.19) [100/8/4], ellipsoid to obovoid, ornamented with warts, light yellowish to light cinnamon in mass, inamyloid, cyanophilic; apiculus rounded, eccentric. *Basidia* 46–67 × 7–9 μm, subcylindrical to clavate, sinuous, hyaline with four sterigmata, sterigmata 6.3–9.5 μm long, basal clamp connections present. *Pleurocystidia* 34–49 × 2.7–5 μm, scattered among and scarcely projecting beyond the basidia, cylindrical to clavate, flexuous, thin-walled, smooth, hyaline, clamped. *Hymenophoral trama* of hyaline, thin-walled, interwoven hyphae. *Pileipellis* composed of thin-walled, frequently branched, tightly interwoven hyphae; hyaline to light yellowish; 2–5 μm wide; inflated in the hyphal termini. *Stipitipellis* of thin-walled, parallel, and interwoven cylindrical hyphae; hyaline to light yellowish; 2–6 μm wide; terminations that are difficult to observe. *Pileus and stipe context* white to light yellowish white; composed of thin-walled, interwoven, hyaline hyphae; 3–7 μm wide; sometimes with embryo-like structure in the hyphal termini but difficult to observe. *Caulocystidia* not observed. *Basal mycelium* smooth, with clamp connections and rosette-like calcium salt crystals on the surface. Clamp connections present in all tissues.

Ecology and habitat: Solitary to scattered on the ground with moss, humus, and debris in mixed broadleaf–conifer forest dominated by *Pinus* L., *Quercus* L., and *Betula* L. [36], sometimes forming obvious fairy ring, elev. 1300–1394 m, currently only known to exist in northwestern China from September to October.

Other specimens examined: China, Shaanxi Province, Yan’an City, Huanglong County, Huanglong Mountains, Caijiachuan Forest Farm, on litter in mixed broadleaf–conifer forest dominated by *P. tabuliformis*, *Q. mongolica*, and *B. pendula* subsp. *mandshurica*; elev. 1330 m; 35°49′27″ N, 109°54′49″ E; 29 September 2022; B.Y. Ma, J.X. Wei, HL202 (HMAS256998); GenBank: ITS = OQ801493; LSU = OQ801491; mtSSU = OQ801495; atp6 = OQ790053; ibid., HL203 (MNWAFU-CF-P209); GenBank: ITS = OQ929929; LSU = OQ929933; mtSSU = OQ929931; atp6 = OQ924518; ibid., HL204 (MNWAFU-CF-P210); GenBank: ITS = OQ929930; LSU = OQ929934; mtSSU = OQ929932; atp6 = OQ924519.

## 4. Discussion

Previous molecular phylogenetic analyses for the cantharelloid, clavarioid, gomphoid, and phalloid fungi were conducted based on the multiple loci of LSU, nuclear small subunit rDNA (SSU), atp6, mtSSU, the second largest subunit of RNA polymerase (RPB2), and translation elongation factor subunit 1a (EF-1a) [1,2,4,37,38]. In our study, three loci of LSU, atp6, and mtSSU were chosen for analyzing the phylogenetic relationships of Gomphales, referring to Giachini et al. [2] and Xu et al. [4]. The results of phylogenetic analyses showed some inevitable topological incongruence and unreliable paraphyletic status of some genera when the three genes were analyzed individually (Supplementary Figure S1–S3). While two or three genes were concatenated, the divergences above were well settled. The phylogenetic analyses based on the combined atp6–mtSSU and LSU–atp6–mtSSU datasets indicated that *Luteodorsum huanglongense* generated an autonomous branch that fit well within Gomphales (Supplementary Figure S4, Figure 1). Thus, *Luteodorsum* was proposed as a novel genus. Instead of being closely related to morphologically similar species from *Gomphus*, *Phaeoclavulina*, and *Turbinellus*, *L. huanglongense* was clustered in a clade with *Gomphocantharellus*, *Ramaria* subg. *Echinoramaria*, Clavariadelphaceae, and Lentariaceae. Interestingly, this clade did not exhibit similar macromorphologies due to the phylogenetic affinity, but instead exhibited a great morphological diversity, including coral, gomphoid, cantharelloid, clavarioid, and resupinate hydnoid mushrooms [1,8,9,10,11,39].

Based on its macromorphology, *L. huanglongense* is easily recognizable as a cantharelloid–gomphoid mushroom in the field due to its typical features of sturdy flesh, fan to funnel shape, and wrinkled outer surfaces (Figure 3). Before our study, five genera of Gomphales were considered as cantharelloid–gomphoid mushrooms, namely *Gomphocantharellus*, *Gloeocantharellus*, *Gomphus*, *Phaeoclavulina*, and *Turbinellus*. Among them, *Gomphocantharellus* and *Gloeocantharellus* are more similar to chantarelle mushrooms due to their distinct gill-like hymenophore (false lamellae). In particular, *Gomphocantharellus* has smooth cylindrical basidiospores and a white spore print, while *Gloeocantharellus* has special gloeocystidia in the hymenium. These above characteristics help to distinguish *Gomphocantharellus* and *Gloeocantharellus* from other gomphoid genera due to their common echinulate or verrucose basidiospores, brownish spore print, and the absence of gleoplerous hyphae, including *Luteodorsum* (Figure 4) [4,19,40,41]. The other three genera, *Gomphus*, *Phaeoclavulina*, and *Turbinellus*, are traditional gomphoid mushrooms typified by wrinkled hymenophore; large and coarse scales on the cap surface; and stipes that are fused together, sharing two or more caps. Among them, *Phaeoclavulina* contains a minority of gomphoid taxa but a majority of representative coral fungi. In contrast to other gomphoid genera, *L. huanglongense* has unipileate basidiomes, an almost glabrous to fibrillose pileus without obvious scales, and prominent separate stipes (Figure 3).

Remarkably, the smooth basal mycelium of *L. huanglongense* was observed to be covered with abundant rosette-like druse crystals (Figure 2B and Figure 4J,K). While some other Gomphales species from *Hydnocristella*, *Clavariadelphus*, *Lentaria*, and *Phaeoclavulina* have also been reported to produce various crystals on the basal mycelium and rhizomorph hyphae, their composition remains unknown [8,9,10,20,42]. The EDS analyses of the druse crystals from *L. huanglongense* confirmed the presence of Ca, C, and O (Figure 2, Table 2). The druse crystals from *L. huanglongense* were found to be similar to spherical aggregates of calcium oxalate (CaOx) formed in mesophyll cells from *Abutilon theophrasti* Medik. and *Acacia robeorum* Maslin, instead of the needle-shaped, prismatic, or flaky CaOx found in multiple white-rotting Agaricomycotina fungi, mycorrhiza fungi, and plant pathogen fungi [8,43,44,45,46,47,48]. Some studies have indicated that the production of CaOx crystals by several oxalate-producing fungi is associated with the extraction of Ca^2+^ from calcium-containing minerals [47,49]. The soil type of the sampling site in a mixed broadleaf–conifer forest dominated by *Pinus*, *Quercus*, and *Betula* was alkaline cinnamon soil [50,51]. In 0–6-, 6–13-, and 13–32-cm cinnamon soil layers, the calcium carbonate (CaCO_3_) content reached 2.00%, 10.51%, and 17.01%, respectively, indicating a relative abundance of Ca^2+^ [52]. It has been suggested that an excess of Ca^2+^ could enhance the CaOx crystal production of fungi [47]; thus, the capability of *L. huanglongense* to produce CaOx suggested that it may play an important role in soil ecology.

Many gomphoid mushrooms have been reported to be edible and form mycorrhizal associations with trees [41,53,54]. Although *L. huanglongense* was found around *Pinus*, *Quercus*, and *Betula* and may have an ectomycorrhizal association with these genera, the evidence as to whether it is edible or has any ecological functions is currently insufficient. Overall, the distinctive features of *L. huanglongense* and its phylogenetic placement in Gomphales make it a unique and interesting addition to the cantharelloid–gomphoid mushroom category. Further research is needed to investigate the edibility and ecological role of *L. huanglongense*.

## Figures and Tables

**Table 1:** Taxa used in phylogenetic analyses, along with their GenBank accession numbers for LSU, atp6, and mtSSU sequence data. “—” indicates that the sequence was unavailable in GenBank. Accession numbers for sequences generated in this study are denoted in boldface.

Fungal Taxon	Specimen Voucher	LSU	atp6	mtSSU
*Beenakia fricta*	K2083	AY574693	AY574833	AY574766
*Clavariadelphus ligula*	OSC67068	AY574650	AY574793	AY574723
*Clavariadelphus lovejoyae*	OSC61068	AY577827	AY577865	AY577854
*Clavariadelphus occidentalis*	OSC37018	AY574648	AY574791	AY574721
*Clavariadelphus truncatus*	OSC67280	AY574649	AY574792	AY574722
*Gautieria caudata*	OSC59201	DQ218483	DQ218767	DQ218658
*Gautieria crispa*	OSC61308	DQ218484	DQ218768	DQ218659
*Gautieria monticola*	OSC65121	AY574651	AY574794	AY574724
*Gautieria pterosperma*	OSC69649	DQ218614	DQ218900	DQ218747
*Gautieria parksiana*	OSC58907	AY574652	AY574795	AY574725
*Gautieria otthii*	REG636	—	EU339254	AF393085
*Gloeocantharellus albidocarneus*	FCME14883	—	MH537976	MT271764
*Gloeocantharellus calakmulensis*	FCME19868	—	MH537977	MT271765
*Gloeocantharellus dingleyae*	PDD:30179	AY574668	—	AY574741
*Gloeocantharellus novae-zelandiae*	PDD:44960	AY574666	AY574809	AY574739
*Gloeocantharellus pallidus*	BPI54917	AY574673	AY574815	—
*Gloeocantharellus papuanus*	PERTH4549	AY574667	AY574810	AY574740
*Gloeocantharellus pleurobrunnescens*	1924	MT261811	MH537978	MT271766
*Gloeocantharellus purpurascens*	TENN12793	AY574683	AY574823	AY574756
*Gloeocantharellus purpurascens*	TENN14265	AY574684	AY574824	AY574757
*Gomphus clavatus*	UPS	AY574665	AY574808	AY574738
*Gomphus clavatus*	OSC97616	AY574664	AY574807	AY574737
*Gomphus clavatus*	OSC97588	AY577836	AY577874	AY577863
*Gomphus clavatus*	OSC97587	DQ218487	DQ218771	DQ218662
*Gomphocantharellus cylindrosporus*	BJTCFM109	OK660766	OK665160	OK660767
*Gomphocantharellus cylindrosporus*	BJTCFM375	OK660768	OK665161	OK660770
*Gomphocantharellus cylindrosporus*	HSA335	OK660772	OK665162	OK660771
***Luteodorsum huanglongense***	**HMAS256997**	**OQ801490**	**OQ790052**	**OQ801494**
***Luteodorsum huanglongense***	**HMAS256998**	**OQ801491**	**OQ790053**	**OQ801495**
***Luteodorsum huanglongense***	**MNWAFU-CF-P209**	**OQ929933**	**OQ924518**	**OQ929931**
***Luteodorsum huanglongense***	**MNWAFU-CF-P210**	**OQ929934**	**OQ924519**	**OQ929932**
*Hydnocristella himantia*	O102156	AY574691	AY574831	AY574764
*Kavinia alboviridis*	O102140	AY574692	AY574832	AY574765
*Lentaria pinicola*	SUCM89	AY574688	—	AY574761
*Lentaria pinicola*	SUCM560	AY574690	AY574830	AY574763
*Lentaria pinicola*	SUCM46	AY574689	AY574829	AY574762
*Phaeoclavulina africana*	TENN39621	AY574653	AY574796	AY574726
*Phaeoclavulina cokeri*	TENN36030	AY574701	AY574843	AY574774
*Phaeoclavulina curta*	OSC8711	AY574713	AY574858	—
*Phaeoclavulina cyanocephala*	TENN37827	AY574710	AY574854	AY574779
*Phaeoclavulina eumorpha*	TENN37842	—	AY574857	AY574782
*Phaeoclavulina eumorpha*	TENN36218	AY574712	AY574856	AY574781
*Phaeoclavulina gigantea*	FH109	AY574703	AY574845	AY574776
*Phaeoclavulina grandis*	BR079158-06	AY574678	AY574820	AY574751
*Phaeoclavulina guadelupensis*	FH120	AY574682	—	AY574755
*Phaeoclavulina guyanensis*	FH84	AY574706	AY574848	—
*Phaeoclavulina insignis*	FH104	AY574704	AY574846	—
*Phaeoclavulina longicaulis*	TENN33826	AY574700	AY574842	AY574773
*Phaeoclavulina ochraceovirens*	OSC23475	AY574714	AY574859	—
*Phaeoclavulina pancaribbea*	TENN31836	AY574707	AY574849	—
*Phaeoclavulina subclaviformis*	BR079159-07	AY574679	—	AY574752
*Phaeoclavulina viridis*	PERTH4302	AY574677	AY574819	AY574750
*Phaeoclavulina viridis*	OSC97708	AY574675	AY574817	AY574748
*Phaeoclavulina viridis*	FH1853	AY574676	AY574818	—
*Ramaria apiculata*	OSC23549	AY574695	AY574836	AY574768
*Ramaria apiculate* var. *brunnea*	TENN53935	AY574696	AY574837	AY574769
*Ramaria araiospora* var. *araiospora*	SUCM739	AF213068	AY574838	AF213141
*Ramaria araiospora* var. *araiospora*	SUCM556	AY574697	AY574839	AY574770
*Ramaria botrytis* var. *botrytis*	SUCM740	AY574699	AY574841	AY574772
*Ramaria botrytis* var. *botrytis*	SUCM457	AY574698	AY574840	AY574771
*Ramaria circinans* var. *anceps*	SUCM615	AY574711	AY574855	AY574780
*Ramaria circinans*	NYS1	AY574702	AY574844	AY574775
*Ramaria rainierensis*	SUCM431	AY574694	AY574835	AY574767
*Ramaria rainierensis*	SUCM231	AF213115	AY574834	AF213135
*Ramaria rubribrunnescens*	SUCM844	AF213098	AY574852	AF213142
*Ramaria stuntzii*	SUCM214	AF213102	AY574850	AF213134
*Ramaria suecica*	BPI1	AY574705	AY574847	—
*Ramaria vinosimaculans*	OSC23287	AY574709	AY574853	AY574778
*Turbinellus floccosus*	MICH5588	AY574660	AY574803	AY574733
*Turbinellus floccosus*	OSC69167	AY574656	AY574799	AY574729
*Turbinellus floccosus*	OSA-MY-1839	AY574654	AY574797	AY574727
*Turbinellus floccosus*	OSA-MY-1840	AY574655	AY574798	AY574728
*Turbinellus floccosus*	TENN33233	AY574657	AY574800	AY574730
*Turbinellus floccosus*	SFSU21238	AY574658	AY574801	AY574731
*Turbinellus floccosus*	TENN33295	AY574659	AY574802	AY574732
*Turbinellus floccosus*	MICH10721	AY574661	AY574804	AY574734
*Turbinellus floccosus*	UC759902	AY574662	AY574805	AY574735
*Turbinellus floccosus*	UC924302	AY574663	AY574806	AY574736
*Turbinellus fujisanensis*	OSA-MY-1841	AY574670	AY574812	AY574743
*Turbinellus fujisanensis*	OSA-MY-1842	AY574669	AY574811	AY574742
*Turbinellus kauffmanii*	OSC97590	AY574672	AY574814	AY574745
*Turbinellus kauffmanii*	MICH10069	AY574671	AY574813	AY574744
*Mutinus elegans*	OSC107657	AY574643	AY574785	AY574717
*Phallus impudicus*	OSC107655	AY574642	AY574784	AY574716
*Pseudocolus fusiformis*	DSH96-033	AF518641	—	AF026666

**Table 2:** The content of different elements in crystals on the surface of the basal mycelium.

Element	Weight%	Atomic%	Weight% (Corrected)	Atomic% (Corrected)
CK	5.83	18.18	6.69	13.58
OK	16.89	39.53	32.41	49.38
PtM	40.32	7.74	—	—
CaK	36.97	34.55	60.90	37.04
Matrix	Correction	ZAF	Correction	ZAF

## Data Availability

Not applicable.

## Supplementary Materials

The following supporting information can be downloaded at: https://www.mdpi.com/article/10.3390/jof9060664/s1, Figure S1: Phylogenetic tree generated from a maximum likelihood analysis based on atp6 sequences, depicting the phylogenetic relationships of Gomphales. *Phallus impudicus*, and *Mutinus elegan* were used as outgroups. The nodes above the branches indicate the maximum likelihood bootstrap support (MLBS) values (≥70%) and maximum parsimony bootstrap support (MPBS) values (≥70%). The branches that presented a Bayesian posterior probability (BPP) ≥0.95 are thicker. The novel sequences are highlighted in bold and orange shade; Figure S2: Phylogenetic tree generated from a maximum likelihood analysis based on mtSSU sequences, depicting the phylogenetic relationships of Gomphales. *Phallus impudicus*, *Mutinus elegans*, and *Pseudocolus fusiformis* were used as outgroups. The nodes above the branches indicate the maximum likelihood bootstrap support (MLBS) values (≥70%) and maximum parsimony bootstrap support (MPBS) values (≥70%). The branches that presented a Bayesian posterior probability (BPP) ≥ 0.95 are thicker. The novel sequences are highlighted in bold and orange shade; Figure S3: Phylogenetic tree generated from a maximum likelihood analysis based on LSU sequences, depicting the phylogenetic relationships of Gomphales. *Phallus impudicus*, *Mutinus elegans*, and *Pseudocolus fusiformis* were used as outgroups. The nodes above the branches indicate the maximum likelihood bootstrap support (MLBS) values (≥70%) and maximum parsimony bootstrap support (MPBS) values (≥70%). The branches that presented a Bayesian posterior probability (BPP) ≥ 0.95 are thicker. The novel sequences are highlighted in bold and orange shade; Figure S4: Phylogenetic tree generated from a maximum likelihood analysis based on combined atp6 and mtSSU sequences, depicting the phylogenetic relationships of Gomphales. *Phallus impudicus*, *Mutinus elegans*, and *Pseudocolus fusiformis* were used as outgroups. The nodes above the branches indicate the maximum likelihood bootstrap support (MLBS) values (≥70%) and maximum parsimony bootstrap support (MPBS) values (≥70%). The branches that presented a Bayesian posterior probability (BPP) ≥ 0.95 are thicker. The novel sequences are highlighted in bold and orange shade.
