# Supplementary material for: Luteodorsum huanglongense (Gomphaceae, Gomphales), a New Genus and Species of Gomphoid Fungus from the Loess Plateau, Northwest China
Source: J Fungi (Basel). 2023 Jun 13;9(6):664. doi: 10.3390/jof9060664 (PMC10304689; doi:10.3390/jof9060664)
Supplement: Supplementary file 1 [file jof-09-00664-s001.zip › jof-2374157-supplementary.pdf]

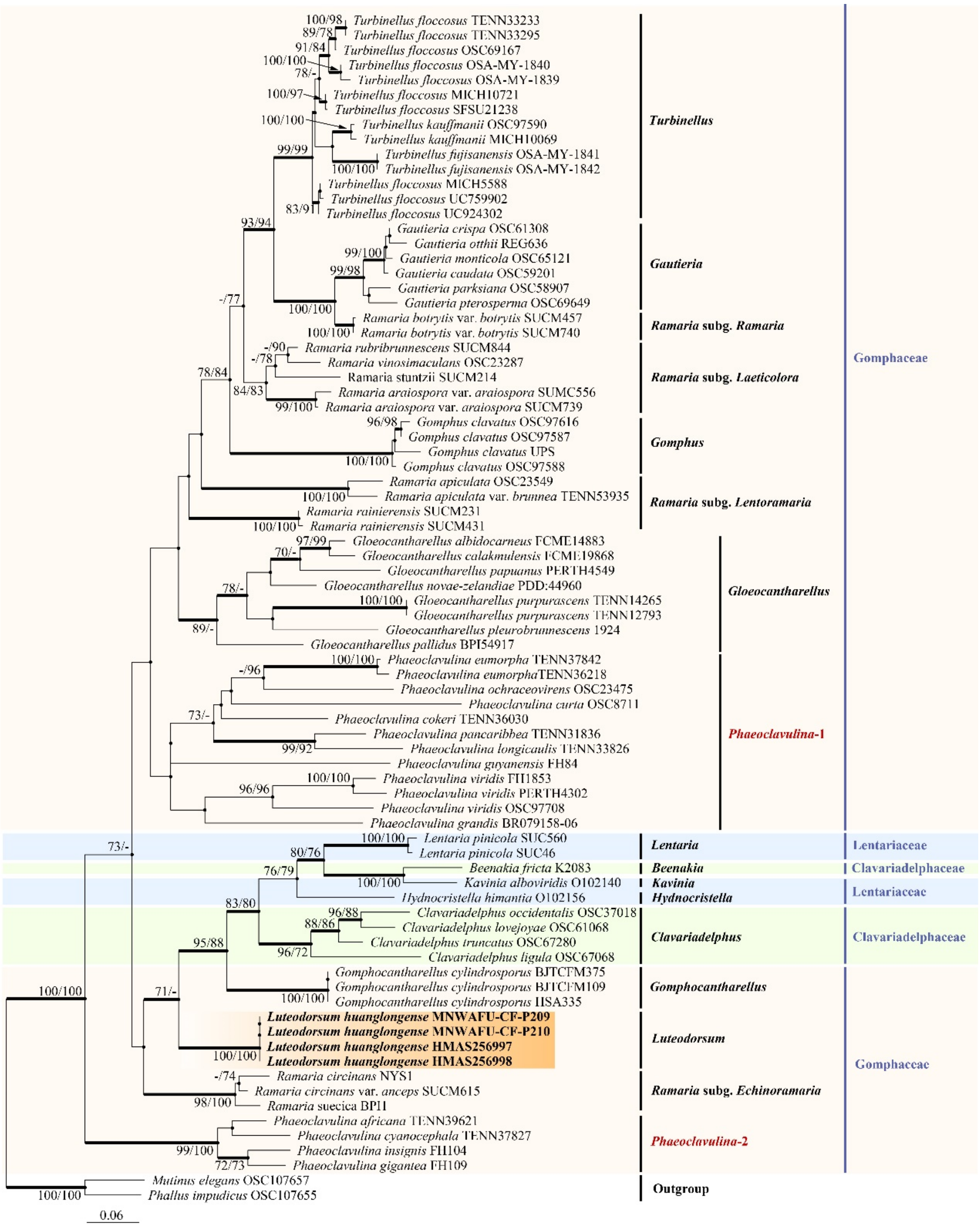

Supplementary Figure S1. Phylogenetic tree generated from a maximum likelihood analysis based on atp6 sequences, depicting the phylogenetic relationships of Gomphales. *Phallus impudicus*, and *Mutinus elegan* were used as outgroups. The nodes above the branches indicate the maximum likelihood bootstrap support (MLBS) values (≥70%) and maximum parsimony bootstrap support (MPBS) values (≥70%). The branches that presented a Bayesian posterior probability (BPP) ≥0.95 are thicker. The novel sequences are highlighted in bold and orange shade.

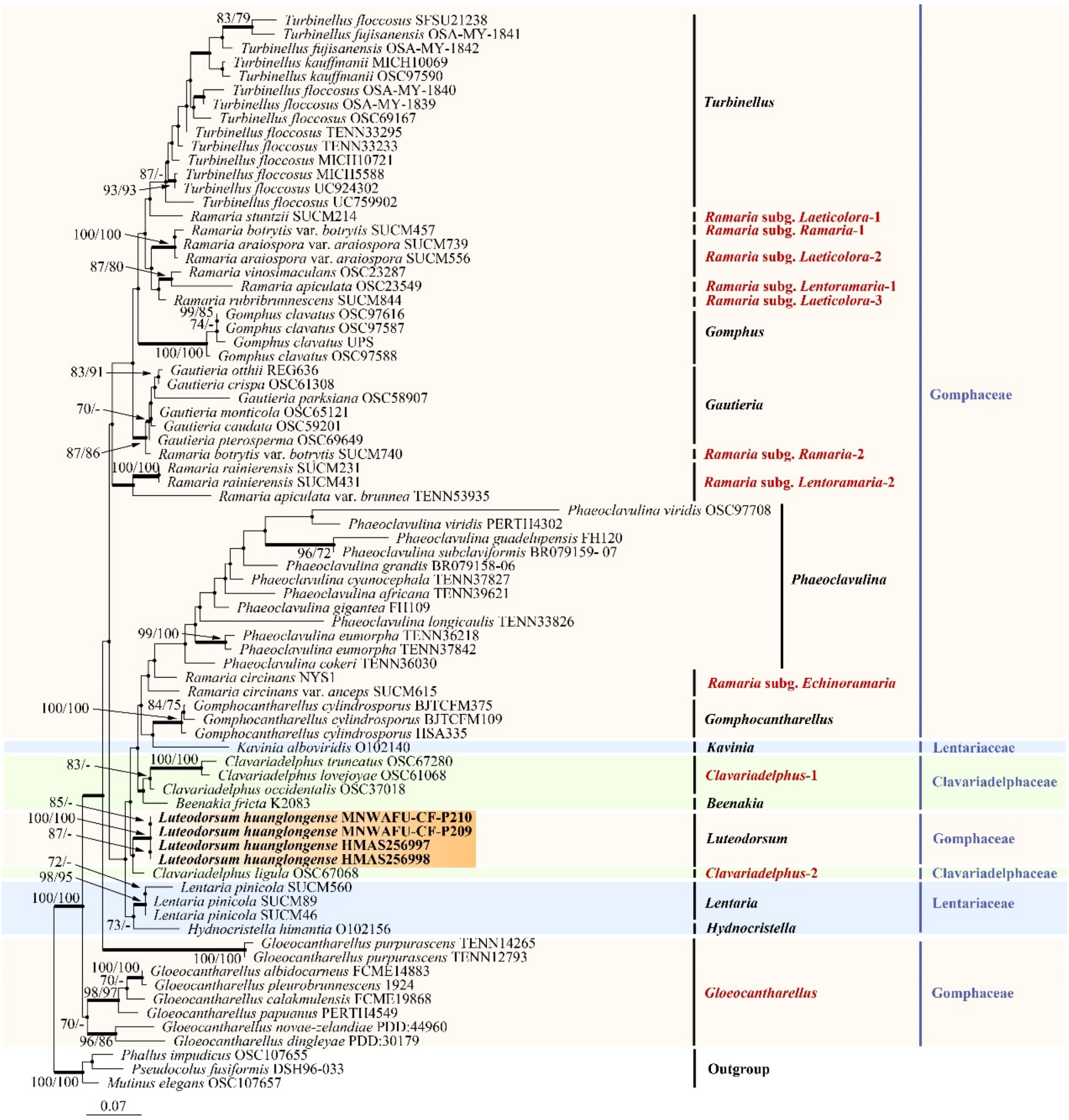

Supplementary Figure S2. Phylogenetic tree generated from a maximum likelihood analysis based on mtSSU sequences, depicting the phylogenetic relationships of Gomphales. *Phallus impudicus*, *Mutinus elegans*, and *Pseudocolus fusiformis* were used as outgroups. The nodes above the branches indicate the maximum likelihood bootstrap support (MLBS) values (≥70%) and maximum parsimony bootstrap support (MPBS) values (≥70%). The branches that presented a Bayesian posterior probability (BPP) ≥0.95 are thicker. The novel sequences are highlighted in bold and orange shade.

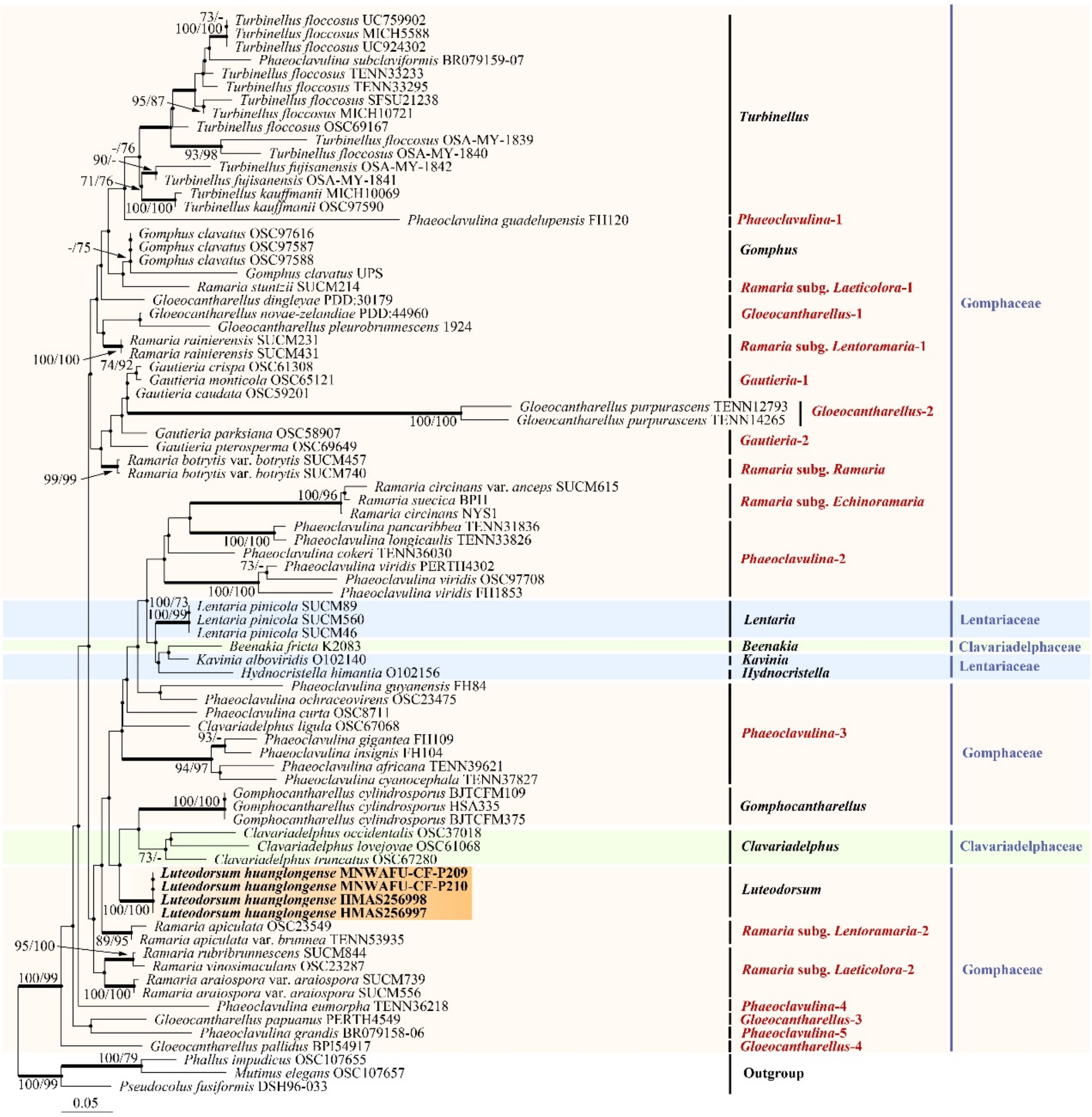

Supplementary Figure S3. Phylogenetic tree generated from a maximum likelihood analysis based on LSU sequences, depicting the phylogenetic relationships of Gomphales. *Phallus impudicus*, *Mutinus elegans*, and *Pseudocolus fusiformis* were used as outgroups. The nodes above the branches indicate the maximum likelihood bootstrap support (MLBS) values (≥70%) and maximum parsimony bootstrap support (MPBS) values (≥70%). The branches that presented a Bayesian posterior probability (BPP) ≥0.95 are thicker. The novel sequences are highlighted in bold and orange shade.

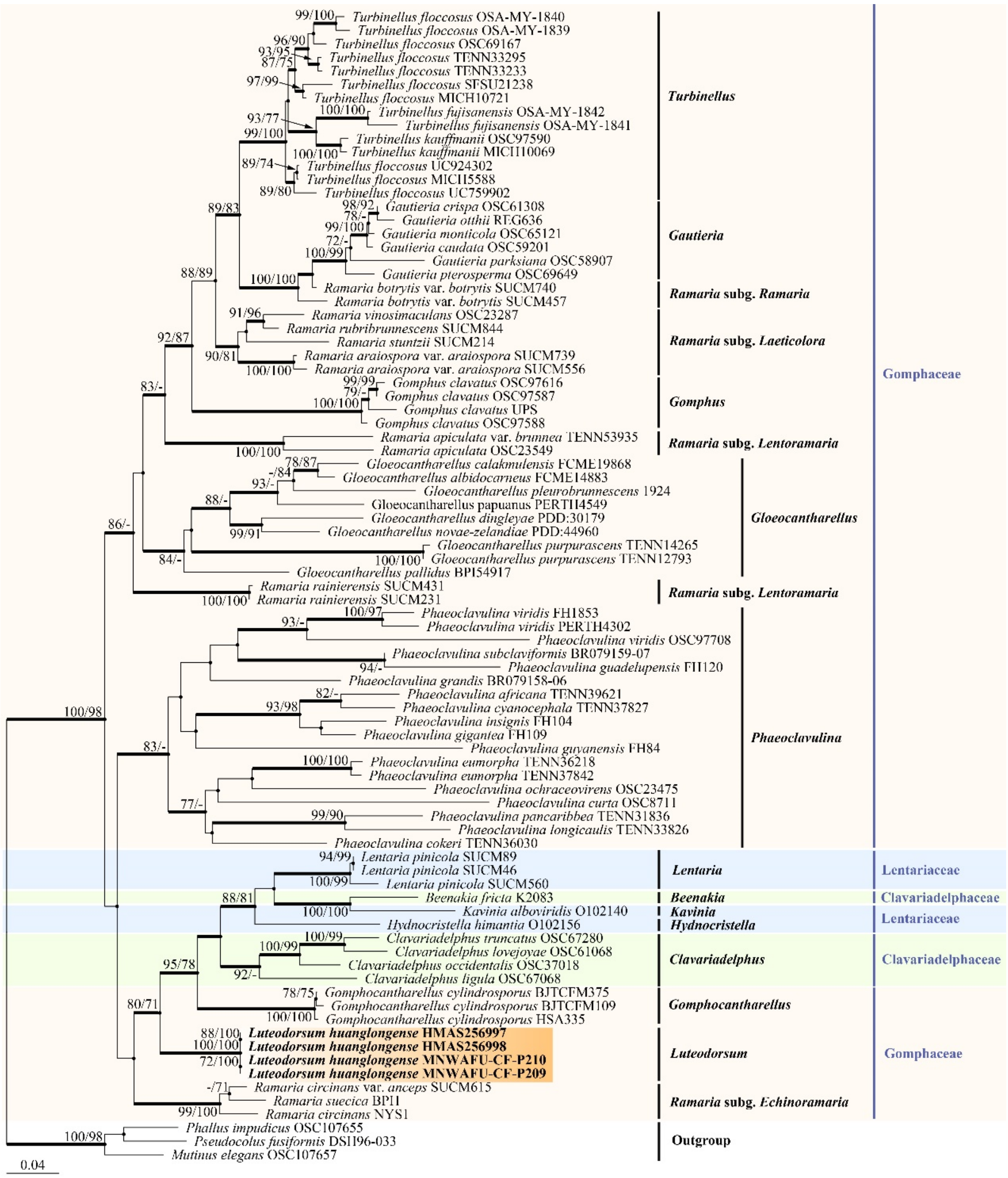

Supplementary Figure S4. Phylogenetic tree generated from a maximum likelihood analysis based on combined atp6 and mtSSU sequences, depicting the phylogenetic relationships of Gomphales. *Phallus impudicus*, *Mutinus elegans*, and *Pseudocolus fusiformis* were used as outgroups. The nodes above the branches indicate the maximum likelihood bootstrap support (MLBS) values (≥70%) and maximum parsimony bootstrap support (MPBS) values (≥70%). The branches that presented a Bayesian posterior probability (BPP) ≥0.95 are thicker. The novel sequences are highlighted in bold and orange shade.
